# Unveiling the Future of Neurorehabilitation: Insights from the 18^th^ Congress from the Society for the Study of Neuroprotection and Neuroplasticity (SSNN) in Conjunction with the WFNR-EFNR Central Asia Regional Meeting

**DOI:** 10.25122/jml-2023-1033

**Published:** 2023-12

**Authors:** Stefana-Andrada Dobran, Alexandra Gherman, Dafin Mureşanu

**Affiliations:** 1 RoNeuro Institute for Neurological Research and Diagnostic, Cluj-Napoca, Romania; 2 Department of Neuroscience, Iuliu Hatieganu University of Medicine and Pharmacy, Cluj-Napoca, Romania

## CURRENT PERSPECTIVES ON NEUROREHABILITATION: ENHANCING PATHWAYS TOWARDS IMPROVED PATIENT CARE

In the domain of neurosciences, neurorehabilitation plays a vital role in the continuum of patient care. As this field evolves, traditional approaches intertwine with groundbreaking technological advancements such as virtual reality systems that facilitate immersive therapy or neurostimulation techniques that promote neural plasticity. This convergence of modern and traditional methods creates a new era of comprehensive and highly effective treatment strategies. Recognizing the intricate nature of neurorehabilitation, specialists operate within dynamic multidisciplinary settings, where perspectives converge to ensure optimal patient outcomes and improved quality of life. Neurologists, physiotherapists, occupational therapists, speech-language pathologists, and other specialists work together to develop tailored interventions and tackle the complex challenges faced by patients. However, the field faces significant limitations, both globally and regionally, which can hinder the delivery of the best care for the patients.

Certain neurological affections, such as stroke, present an increased prevalence, morbidity, and mortality. Therefore, they require more in-depth research and multidisciplinary approaches, due to the interplay of genetic, behavioral, and systematic factors that contribute to their development and the associated complications. Consequently, there is a pressing need for targeted action to increase education and awareness and develop capacity to enhance neurorehabilitation.

## UNIFYING NEUROREHABILITATION: THE WFNR-EFNR CENTRAL ASIA REGIONAL MEETING

Following the Dysphagia Course Series, which took place between June 22^nd^ and 23^rd^ June 2023, the WFNR-EFNR Central Asia Regional Meeting unfolded on the 24^th^ of June in Tashkent, Uzbekistan, serving as a dynamic platform for the exchange of knowledge, groundbreaking research, and innovative practices in neurorehabilitation.

**Figure 1:**
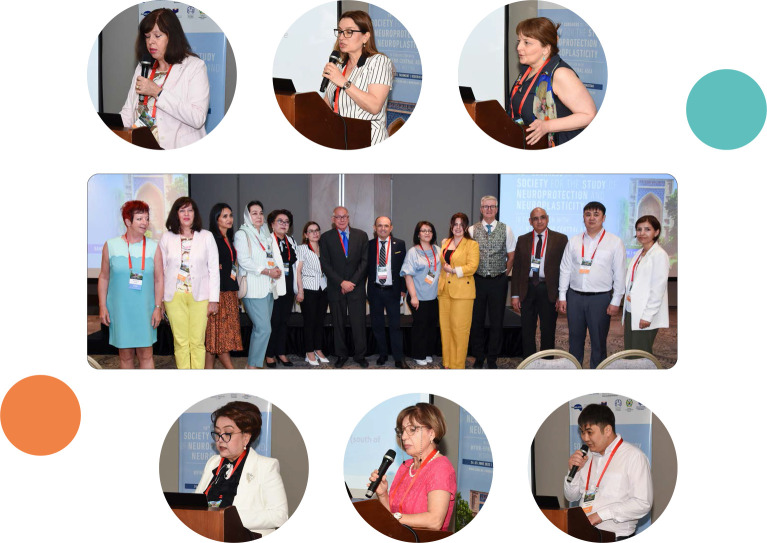
The WFNR-EFNR Central Asia Regional Meeting: Speakers and Country Representatives

Highlighting the significance of collaboration, the WFNR-EFNR High-Level Meeting aimed to expand the outreach initiative of both organizations and foster the development of neurorehabilitation services in a collaborative context. Comprising a network of esteemed professionals with a shared interest in neurorehabilitation, the Regional Meeting also featured the perspectives of country representatives (Figure 1) to enhance understanding of the current landscape of neurorehabilitation services. The European Federation of Neurorehabilitation Societies (EFNR) and the World Federation for Neurorehabilitation (WFNR) operate as a synergic complex, with a common goal to amplify the quality of neurorehabilitation services and offer a better understanding of the field. Through promoting cooperation strategies at international and regional levels, the regional meeting was committed to investigating ways in which the two federations can provide support and aid in improving services. A particular focus concerned the current state of stroke services, especially acute rehabilitation.

Stroke is a multifaceted problem that can affect individuals across different age groups. Among young adults, strokes are associated with congenital heart defects, hereditary arterial hypertension, blood diseases, and substance misuse. Conversely, in the older population, strokes are often correlated with chronic conditions and behavioral factors, such as smoking.

**Figure 2:**
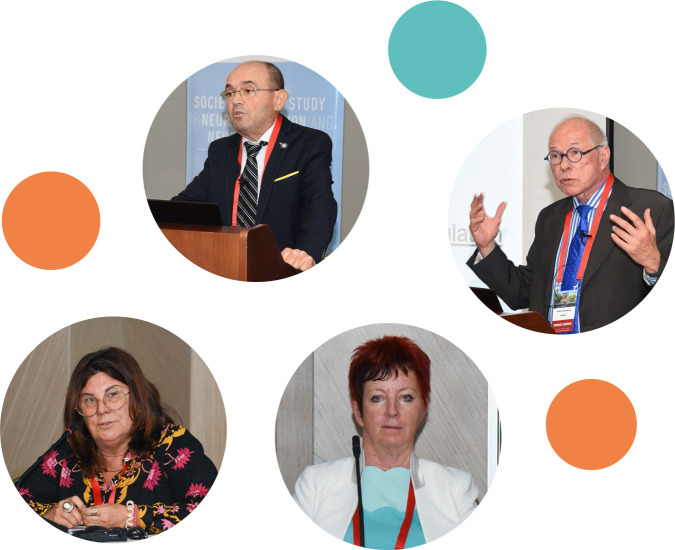
The High-Level Meeting Presidium (Prof. Dafin Muresanu, Prof. Volker Homberg, Prof. Caterina Pistarini, Dr. Dana Boering)

The event featured country presentations from Uzbekistan, Kazakhstan, Georgia, Armenia, Azerbaijan, Kyrgyzstan, and Belarus, approaching the current state of healthcare. Furthermore, the discussion extended to explore avenues for growth and innovations, involving all the participants, concluding with the identification of future strategies and tackling forthcoming guidelines. The Presidium of the High-Level Meeting brought together Prof. Volker Hömberg (Germany), WFNR President, Prof. Dafin F. Mureşanu (Romania), EFNR President, Dr. Dana Boering (Germany), EFNR Secretary, and Prof. Caterina Pistarini (Italy), WFNR Secretary (Figure 2). Renowned specialists from various countries participated in the meeting to share insights into current practices and limitations, engaging in discussions about future avenues for improvement. Among the esteemed speakers were Prof. Yakuthon Madjidova (Uzbekistan), Yerzhan Boranbayevich Adilbekov (Kazakhstan), Nino Lobjanidze (Georgia), Laura Movsisyan (Armenia), Sadagat Huseynova (Azerbaijan), Chyngyz Niyazbekov (Kirghizstan), Sidyakina Irina Vladimirovna (Russia), and Ludmilla Anatskaia (Belarus).

The meeting focused on the current state of stroke services, addressing epidemiology, availability and accessibility of stroke units, and the presence of specialized healthcare professionals. The speakers explored key challenges and gaps in stroke services, and addressed measures and initiatives under implementation, with a particular emphasis on the role of the federations in providing support. The discussions also revolved around the timing and intensity of rehabilitation interventions, the use of multidisciplinary teams, and the incorporation of specialized technological approaches. Moreover, the speakers exchanged insights on general aspects of neurorehabilitation, such as commonly used interventions and therapies for stroke recovery in each country, and shared unique programs or resources available to promote long-term neurorehabilitation and enhance the quality of life for stroke patients.

As the speakers discussed country-specific challenges, some crucial limitations in the stroke healthcare systems were highlighted, such as the:

- lack of available stroke therapies (e.g., thrombolysis and mechanical thrombectomy);
- absence of national networks for organized stroke care;
- lack of primary stroke prevention;
- limited funding for costly interventions;
- discrepancies in the development of stroke medical care and rehabilitation;
- shortage of acute rehabilitation options to prevent secondary complications;
- scarcity of neurorehabilitation departments and specialists;
- limited access to thrombolytic therapy in public hospitals;
- shortage of stroke units in public clinics ;
- lack of a multidisciplinary approach to stroke care;
- shortage of specialists (e.g., occupational therapists);
- insufficient hospital beds to meet the population's needs;
- absence of standardized neurocognitive rehabilitation;
- inadequate in-hospital reimbursement;
- limited availability of imagistic tools in state-owned facilities;
- scarcity of fully equipped stroke departments;
- limited access to thrombolytic drugs in public hospitals;
- delayed ambulance calls;
- long distances to specialized vascular centers;
- insufficient training of medical staff.

Examples of good practices described during the meeting included robust state support for individuals with disabilities, encompassing pensions, privileges, free university education, and even free rehabilitation treatment for a specified duration. Certain initiatives, such as the National Stroke Program (NSP) in Armenia and the Austrian Rehabilitation Exchange program with Uzbekistan (AUREUS) stand as a cornerstone to improving patient care by developing capacity and improving knowledge. A noteworthy collaboration between Belarus and Austria has been established to elevate the quality of acute and subacute stroke neurorehabilitation in accordance with European standards.

To address the existing challenges, intertwining bottom-up and top-down approaches to ensure better functioning of the medical systems, as well as improving knowledge and prevention practices in the population through primary care is needed.

## POLICY, MANAGEMENT, AND COLLABORATION

A critical focus during the presentations targeted building robust medical infrastructure and establishing comprehensive stroke centers. Improving communication channels between ministries of health and other institutions as well as policy initiatives to attract young physicians and nurses to the field of stroke care, ensuring a sustainable workforce for the future, is of utmost importance. Moreover, long-term collaboration with international stroke experts and institutions should be fostered to ensure knowledge and expertise exchange. It is important to enhance access to early rehabilitation by improving regulatory legal acts and ensuring better availability of necessary equipment. Moreover, using technological aids in the delivery of medical services, such as telestroke programs, can further improve access to care for various demographics.

## EDUCATION AND TRAINING

Introducing specialized training courses for professionals involved in acute and subacute in-hospital patient care, along with interdisciplinary training programs can significantly contribute to improved stroke rehabilitation outcomes. Online healthcare degrees and certificates can represent one avenue for facilitating continuous professional development. Furthermore, training for specific personnel, such as nurses, on specific aspects of neurorehabilitation (e.g., dysphagia management, early mobilization, and early rehabilitation skills) could aid in building capacity for the medical system. Raising awareness and educating the general population on the impact and prevention of stroke and other neurological affections as a measure of primary prevention is a crucial step that can be achieved through educational events, and campaigns, and aid in reducing the burden on the medical system and improving life quality.

## EMPOWERING NEUROREHABILITATION: INSIGHTS FROM THE 18^TH^ CONGRESS FROM THE SOCIETY FOR THE STUDY OF NEUROPROTECTION AND NEUROPLASTICITY (SSNN)

**Figure 3:**
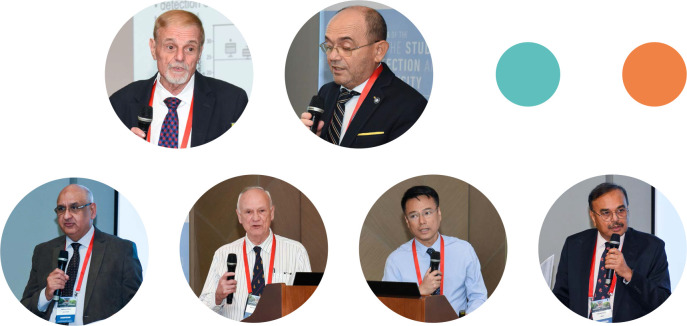
Speakers from the 18^th^ SSNN Congress

With a particular focus on stroke care, including acute rehabilitation, the 18^th^ Congress of the Society for the Study of Neuroprotection and Neuroplasticity (SSNN) delved into a wide array of fundamental and practical aspects of neurorehabilitation. Esteemed experts from Kazakhstan, Belarus, USA, Israel, Germany, Austria, Azerbaijan, Vietnam, Georgia, Romania, India, Italy, and Abu Dhabi actively participated in the event sharing their expertise with participants to improve the field of neurorehabilitation (Figure 3).

The congress commenced with a warm welcome from the local host, Prof. Yakuthon Madjidova (Uzbekistan), Prof. Volker Hömberg (Germany), Prof. Dafin F. Mureşanu (Romania), and Prof. Natan Bornstein (Israel). The first session, chaired by the latter three, featured insightful presentations on pharmacology, post-stroke cognitive decline, and family-based rehabilitation.

Prof. Dafin F. Mureşanu (Romania) explored the EFNR-WFNR perspective on brain recovery in the context of the evolving nature of stroke therapeutic approaches, particularly focusing on thrombolytic therapy and its impact. Considering the high mortality and morbidity rates among stroke patients, rehabilitation and biological reserve play a crucial role in determining the brain's capacity for resilience to various lesional mechanisms. Professor Mureşanu highlighted the dedicated efforts of EFNR and WFNR in strengthening disability prevention measures, working towards enhancing brain recovery outcomes and optimizing patient well-being.

Prof. Volker Hömberg (Germany) delved into the crucial role of pharmacological agents in neurorehabilitation, discussing certain drugs that can aid in facilitating brain recovery and reducing impairment, cautioning against the use of substances that may hinder brain repair and discussing the potential impact of antidepressants in neurorehabilitation. Prof. Hömberg discussed drugs that influence states of diminished consciousness and the relevance of multimodal drugs for post-stroke acute treatment.

Prof. Natan Bornstein’s (Israel) talk was centered upon the Tel-Aviv Brain Acute Stroke Cohort (TABASCO), an important initiative aimed at characterizing inflammatory, stress, and neuroimaging biomarkers that can help predict and detect vulnerable individuals with risk of stroke. This research holds the potential to shape new concepts for early interventions and novel treatment strategies for those at higher risk. Prof. Bornstein emphasized that the combination of behavioral therapies and safe, effective pharmacological adjuvants can significantly enhance and promote brain recovery after stroke, particularly in cases involving cognitive impairment.

Prof. Nirmal Surya (India) emphasized the significance of family-based rehabilitation, particularly in developing countries where neurological care delivery and medical personnel availability may differ, leading to shortcomings. The financial burden on patients and healthcare systems is a considerable percentage of developing countries lacking neurorehabilitation services, while others face challenges with the quality and structure of their existing systems. Prof. Surya advocated for community-based and domiciliary-tailored interventions to facilitate community reintegration during the recovery phase. The intimate social system and family structure play a pivotal role in providing physical, emotional, and spiritual support, helping individuals overcome the effects of disability, reducing costs, and improving their quality of life. Moreover, the use of technology can be employed to enhance the effectiveness of family-based rehabilitation. Embracing family-centered approaches can prove transformative, offering a more sustainable and comprehensive solution for neurological care in developing countries.

The following session, skillfully moderated by Prof. Francesca Pezzella (Italy) and Prof. Sabahat Wasti (Abu Dhabi), delved into the Stroke Action Plan for Europe, hyperacute stroke rehabilitation, and dysphagia management.

Dr. Sabahat Wasti (United Arab Emirates) discussed hyperacute stroke rehabilitation, signaling a need for more robust and universally standardized care in this area. Dr. Wasti emphasized the importance of integrating multidisciplinary concepts into stroke rehabilitation and proposed a model currently under development for multidisciplinary stroke rehabilitation aiming to provide comprehensive and effective rehabilitation care for stroke patients in the hyperacute phase and beyond.

Prof. Stefanie Duchac (Germany) presented evidence-based practices in dysphagia management, highlighting the crucial role of evidence in guiding effective interventions. Prof. Stephanie Duchac emphasized the significance of external, internal, and social evidence in shaping evidence-based practices, highlighting that each type of evidence carries equal importance but may have different focuses depending on the stage of rehabilitation. Furthermore, Prof. Duchac discussed the latest developments in rehabilitation research, pinpointing on current approaches and international findings that advance dysphagia management. Overall, her presentation focused on evidence-based practices, the challenges faced in dysphagia management, and the ongoing efforts to improve patient outcomes through research and international collaboration.

Mr. Björn Degen (Austria) delved into post-stroke dysphagia and dysphagia in neurodegenerative diseases, underscoring the scarcity of guidelines for dysphagia management in neurodegenerative conditions, despite its profound implications for mortality, quality of life, and healthcare costs. The presentation provided valuable insights into the fundamental steps and levels of dysphagia management, while also addressing its limitations. Mr. Björn Degen outlined the process, starting from screening to clinical and instrumental assessments, describing available tools and their importance in achieving optimal outcomes. The discussion emphasized the significance of appropriate assessment and interventions in improving patient well-being and overall healthcare outcomes.

The second day began with the third session, chaired by Prof. Nirmal Surya (India) and Prof. Yakuthon Madjidova (Uzbekistan), which explored the organization of neurorehabilitation programs within ESO-EAST countries, the WFNR perspective on future neurorehabilitation, neurogenic dysphagia, and the interplay of Artificial Intelligence (AI) with neurosciences.

Mr. Axel Kohlmetz’s (Austria) presentation approached the ESO-EAST (European Stroke Organisation – Enhancing Acute Stroke Treatment) project. He elaborated on the Stroke Action Plan-Europe (SAP-E) and other ESO initiatives like RES-Q, emphasizing the necessity for country-specific projects to address treatment gaps and ensure progress towards the 2030 goals when SAP-E concludes. Mr. Axel Kohlmetz also shared examples of impactful rehabilitation projects and outlined implementation plans to pave the way for meaningful advancements in stroke treatment and care.

Prof. Volker Hömberg (Germany) explored the significant advancements in neurological rehabilitation over the past years, emphasizing the importance of a deeper understanding of the underlying neurological restorative processes to develop more precise and effective strategies. Professor Hömberg touched upon the promising developments in multimodal drugs and the relevance of biometric analyses in neurological rehabilitation. Overall, his presentation underscored the importance of tailored, targeted approaches to optimize patient outcomes.

Prof. Caterina Pistarini (Italy) delved into the topic of neurogenic dysphagia, a neurological dysfunction that leads to swallowing disorders and can disrupt the sensorimotor function during the oral and pharyngeal phases of swallowing. Prof. Pistarini provided an in-depth analysis of the existing body of literature, with findings suggesting promising potential for new applications of certain maneuvers in dysphagia treatment. Although the evidence remains inconclusive, this opens up numerous research avenues that call for further exploration in the field of dysphagia.

Dr. Russel Andrew (USA) presented to the audience an intriguing interplay between the human mind and Artificial Intelligence, drawing insights from science and literature, via a past-present dichotomy. He explored various historical perspectives on AI's ability to replicate human intellect, taking into account recent advancements in AI, robotics, and neuroscience. According to Dr. Andrews, the crux of the matter lies in how these 'mechanical' and 'human' aspects converge, giving rise to the brain-mind dilemma, which could be addressed through a 'diplomatic scientific resolution'. By exploring the similarities and intersections between AI and human cognition, this approach seeks to unravel the complexities of the human mind and AI, paving the way for potential solutions and advancements.

Moving on to the fourth session, chaired by Prof. Caterina Pistarini (Italy) and Dr. Russell Andrews (USA), the pursuit, Austrian rehabilitation exchange with Uzbekistan and the AVANT program in Vietnam were discussed along with the crucial role of motivation in rehabilitation after brain injury.

Prof. Yakuthon Madjidova (Uzbekistan) discussed the collaborative program Austrian Rehabilitation Exchange with Uzbekistan (AUREUS) initiated in 2018 to address crucial needs in the Uzbek post-stroke rehabilitation system. Junior neurologists from Uzbekistan undergo comprehensive training in acute and subacute stroke rehabilitation management at Austrian stroke facilities in a multidisciplinary setting. Upon returning to Uzbekistan, these specialists organize train-the-trainer programs across the country, working closely with the Ministry of Health of the Republic of Uzbekistan. The presentation showcased the positive outcomes achieved so far, with the program receiving the esteemed award of the "Best Project of 2020" by the Health Ministry in the Republic of Uzbekistan. The progress of AUREUS is encouraging and reflects its impactful contribution to improving stroke rehabilitation in Uzbekistan.

Dr. Luong Thuan Khan's (Vietnam) presentation highlighted the AVANT program (Austrian Vietnamese Advancement Neurorehabilitation Treatment), introduced in 2017, to address the growing impact of stroke in Vietnam and the shortcomings in the existing rehabilitation services. This program is based upon the collaboration of doctors, professors, and rehabilitation experts from various centers who provide training to healthcare workers, patients, and caregivers in Vietnam. Remarkably, this comprehensive program has been implemented across 800 hospitals and healthcare centers, making a significant impact on the accessibility and quality of neurorehabilitation services in Vietnam.

Dr. Dana Boering (Germany) delivered a lecture on the vital role of motivation in post-stroke neurorehabilitation. Recognizing the multifaceted challenges patients face, from neurobiological, psychological, social, and clinical factors, Dr. Boering advocated for a deeper integration between the fields of biology and psychology. Her lecture explored the impact of behavioral and pharmacological interventions on motivation to construct a comprehensive framework of multidisciplinary motivation, underlining the significance of motivation assessment and the implementation of motivational tools in clinical practice. By nurturing and enhancing motivation, patients are empowered to actively participate in their rehabilitation, leading to better outcomes and enriching their overall quality of life.

**Figure 4:**
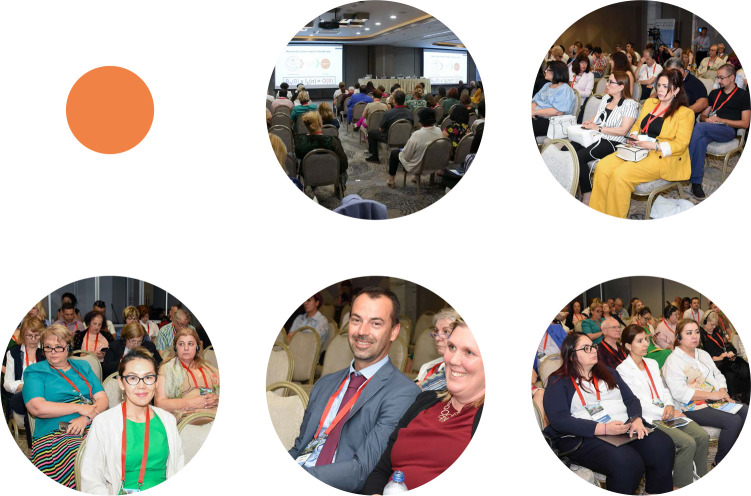
Pictures from the 18^th^ Congress from the Society for the Study of Neuroprotection and Neuroplasticity (SSNN)

Engaging discussions resulted from the sessions, providing a platform for professionals to exchange knowledge and explore innovative approaches in neurorehabilitation. Overall, the scientific event (Figure 4) paved the way for empowering advancements and collaborations, ultimately driving the progress of neurorehabilitation practices worldwide.

## THE VISION OF WFNR-EFNR: ADVANCING NEUROREHABILITATION RESEARCH, CARE, AND EDUCATION

As the prevalence of neurological disorders continues to rise, adopting new multidisciplinary approaches to neurorehabilitation is crucial. Collaboration among national and international societies, as well as specialists, plays a pivotal role in driving progress and stands as the heart of revolutionizing health and care for the population. Moreover, disseminating knowledge and expertise as well as training the new generations of specialists is the cornerstone for building better and more systematic approaches to address common limitations. The ultimate goal is to enhance not only health and medical outcomes but also the quality of life for patients, thereby creating healthier communities and alleviating the burden of disease on a local, national, and global scale. By integrating bottom-up and top-down strategies, gaining diverse perspectives, and developing practical skills, professionals can effectively address the challenges in this fast-paced and demanding field. It is essential to have clinical knowledge with an understanding of emerging technologies and collaborative skills to become well-rounded experts capable of both solving clinical cases and advancing the field of neurotraumatology.

The SSNN, EFNR, and WFNR work hand in hand to cultivate an environment that encourages innovation, collaboration, and lifelong learning in a multidisciplinary setting. Their joint efforts aim to improve the health of individuals and equip specialists with the necessary tools to tackle the present and future challenges in neurorehabilitation. Join us in our pursuit of improving health and care worldwide!

